# Preparation of a novel Fe_3_O_4_/HCO composite adsorbent and the mechanism for the removal of antimony (III) from aqueous solution

**DOI:** 10.1038/s41598-019-49679-9

**Published:** 2019-09-10

**Authors:** Jun Zhang, Ren-jian Deng, Bo-zhi Ren, Baolin Hou, Andrew Hursthouse

**Affiliations:** 1 School of Civil Engineering, Hunan University of Science and Technology, Xiangtan, 411201 China; 2 Hunan Jing Yi Environmental Protection High Tech Development Co. Ltd., Xiangtan, China; 3 School of Computing, Engineering & Physical Sciences, University of the West of Scotland, Paisley, PA1 2BE UK

**Keywords:** Pollution remediation, Environmental chemistry

## Abstract

A novel adsorbent (Fe_3_O_4_/HCO) was prepared via co-precipitation from a mix of ferriferrous oxide and a Ce-rich waste industrial sludge recovered from an optical polishing activity. The effect of system parameters including reaction time, pH, dose, temperature as well as initial concentration on the adsorption of Sb(III) were investigated by sequential batch tests. The Sb(III)/Fe_3_O_4_/HCO system quickly reached adsorption equilibrium within 2 h, was effective over a wide pH (3–7) and demonstrated excellent removal at a 60 mg/L Sb(III) concentration. Three isothermal adsorption models were assessed to describe the equilibrium data for Sb(III) with Fe_3_O_4_/HCO. Compared to the Freundlich and dubinin-radushkevich, the Langmuir isotherm model showed the best fit, with a maximum adsorption capacity of 22.853 mg/g, which exceeds many comparable absorbents. Four kinetic models, Pseudo-first-order, Pseudo-second-order, Elovich and Intra-particle, were used to fit the adsorption process. The analysis showed that the mechanism was pseudo-second-order and chemical adsorption played a dominant role in the adsorption of Sb(III) by Fe_3_O_4_/HCO (correlation coefficient R^2^ = 0.993). Thermodynamic calculations suggest that adsorption of Sb(III) ions was endothermic, spontaneous and a thermodynamically feasible process. The mechanism of the adsorption of Sb(III) on Fe_3_O_4_/HCO could be described by the synergistic adsorption of Sb (III) on Fe_3_O_4_, FeCe_2_O_4_ and hydrous ceric oxide. The Fe_3_O_4_/HCO sorbent appears to be an efficient and environment-friendly material for the removal of Sb(III) from wastewater.

## Introduction

Antimony(Sb) has the properties of both metallic and non-metallic elements^1,2^, and mainly occurs in the form of Sb(III) and Sb(V) oxides in water^1^. However, the lower oxidation state of Sb is highly toxic, for example the toxicity of Sb (III) is 10 times that of Sb (V)^1^. The long-term intake of excess antimony is a serious health risk which could include direct liver and lung damage, damaged the immune system as well as induced cancer. It has subsequently been ranked as one of the high-priority pollutants by USEPA (the US Environmental Protection Agency). The European Union as well as China set 5 μg/L as the maximum allowable concentration of Sb in drinking water, and the USEPA a value of 6 μg/L^3,4^. However, Sb is also important raw material used for example in flame retardants, alloys, ceramics, and glassware. The wide application of Sb in various industries has resulted in an increase in concentration of Sb in the aquatic environment^2,5^. In addition, pollution associated with its extraction and refining is excessive and needs to be addressed in some mining areas^2,5,6^.

Many techniques have been explored for removal of antimony from aqueous systems, including coagulation and precipitation^7^, solidification^8,9^, ion-exchange^10^, membrane separation technology^11^, and electrochemical deposition^12^. Among these adsorption technology had received the greatest attention because of a number of advantages, such as relatively low-cost, high-efficiency, simplicity and eco-friendly^2,13^. Recently, many single adsorbents have been evaluated^8,14–16^. However, there are a number of shortcomings with their application, such as low adsorption capacity and difficulty in separating materials from the aqueous solution when they are saturated^2,5,6^. As frequently used as adsorbents, iron-based materials combine adsorption properties with useful magnetic properties. This makes the separation of solids from liquid phases straightforward using an external magnetic field and allows for recovery and reuse after regeneration^17,18^. The advantage of magnetic separation of adsorbents is a more effective approach to solid-liquid separation than normal separation methods such as sedimentation and filtration^19,20^. Also, magnetic separation is particularly useful when the aqueous solution contains nonmagnetic solid residues as would be found in complex waste water systems^19,21,22^.

The Fe_3_O_4_ adsorbent system has previously been systematically studied as an excellent magnetic material^23^. However, it has a significant limitation in its lower sorption capacity restricting effective application^17^. Synthesis of iron-containing bimetal oxides can greatly enhance adsorbent performance by increasing the amount of surface pores, hydroxyl groups, and tunable surface charge^2,17,18^. Also, earlier studies have found that a Cerium-doped Fe_3_O_4_ magnetic adsorbent tends to have high affinity surface hydroxyl groups and very promising adsorption capacity^17,24–26^. And is considered to be an excellent adsorbent for antimony removal^17,27^. However, cerium is a rare metal widely used in modern devices and is expensive, limiting its application in adsorption systems. The polishing sludge identified, is particularly rich in condensed Cerium (Ce), mostly as hydrous ceric oxide (HCO: CeO_2_·nH_2_O, also known as cerium hydroxide), and is a common component in chemical mechanical polishing (CMP) process wastewaters treatment by product, appearing also as a residue from the liquid crystal display (LCD) industry. China produces more than 5.0 × 10^4^ tons a year according to incomplete statistics. The preparation of the sorbent using Fe_3_O_4_ and the Ce-rich waste, whilst adding additional synthesis steps, uses lower amount of Ce than Cerium-doped Fe_3_O_4_ magnetic adsorbent reported in previous research^17,28^. In addition the sludge is stable and can be produced in a range of particle sizes^29^. The preparation of a successful sorbent based on Fe_3_O_4_/HCO also contributes to the aims of the “2030 Agenda for Sustainable Development”, by reducing the generation of waste residues through recycling and the efficient use of secondary resources^2^. Additionally, the preparation and optimization of Fe_3_O_4_/polishing sludge adsorbent seem to be a key-role to control the removal efficiency of antimony by adsorption. To the best of our knowledge, the mechanism for antimony adsorption by Cerium-doped Fe_3_O_4_ magnetic adsorbent is unclear^17,27^.

In this work, we focus on: (1) synthesis of Fe_3_O_4_/HCO by a modified coprecipitation method, and subsequent detailed characterization; (2) sorption of aqueous Sb(III) by Fe_3_O_4_/HCO and evaluation of capacity and the effects of pH, reaction time, the amount of adsorbent, reaction temperature and initial concentration; (3) tests of models of isothermal adsorption, reaction kinetics and thermodynamics leading to proposals for the mechanism for the adsorption of Sb(III) on Fe_3_O_4_/HCO. We believe this is the first report of the successful preparation of iron-based adsorbents with polishing sludge and its initial application addressing a pressing environmental issue of Sb(III) contamination.

## Experimental

### Synthesis and characterization of Fe_3_O_4_/HCO adsorbent

Polishing sludge material was collected from the wastewater treatment plant of Lansi Technology (Hunan) Co., Ltd. The air-dried bulk analysis (w/w) was: moisture content of sludge is 80.5%, and the other main components are cerium oxide (7.8%), silicon dioxide (4.5%), aluminium oxide (3.8%), calcium oxide (2.8%) and other material (0.6%).

The Fe_3_O_4_/HCO adsorbent was prepared using the following modified coprecipitation method^17,30^. Firstly, a 1000-ml three-necked flask was purged with nitrogen for 10 min and 10 g air dried polishing sludge was added followed by 50 ml of an aqueous solution containing 5.56 g FeSO_4_·7H_2_O and 50 ml of an aqueous solution with 10.8 g FeCl_3_. Secondly, the flask was placed in a water bath at 60 °C and 200 ml 7% aqueous ammonia solution slowly added whilst being agitated at 350 rpm. Thirdly, after continuous stirring for 2 h under the nitrogen atmosphere, the resulting slurry was separated by rapid centrifugation, decanted and washed with deionized water and ethanol followed by drying at 80 °C for 24 h. Finally, the dry mixture was ground into fine powder with a mortar and pestle to pass a 100-mesh sieve, and then used for Sb(III) removal studies.

Particle morphology and crystallinity of Fe_3_O_4_/HCO were characterized using scanning electron microscope (SEM, JSM-6380LV, JEOL Ltd.) and X-ray diffraction (XRD) patterns (D8 Advance, Brook AXS Ltd., Germany). Elements on the surface of Fe_3_O_4_/HCO were analyzed using Energy Dispersive Spectrometer (EDS) (Bruker XFlash 5010, Germany). The XRD was used to identify compounds present in the solid sorbent before and after adsorption of Sb(III). X-ray photo-electron spectroscopy (XPS) spectra focused on Ce, Fe and Sb sorbed onto the Fe_3_O_4_/HCO using a PHI 5000 Versa probe system (Thermo Scientific: Esala 250Xi). All the binding energies were associated with the C 1s peak at 285.1 eV and XPS peak fit version 4.1 was used to analyze the spectral data. N_2_ adsorption-desorption isotherms were used to test the surface area and the pore structures of Fe_3_O_4_/HCO. The specific surface area, pore volume as well as pore diameter of Fe_3_O_4_/HCO were measured by N_2_ adsorption at 77 K using a QuadraSorb Station 1 Instruments (Anton Paar GmbH).

### Adsorption experiments

The influence of experimental variables on the adsorption isotherm and kinetics were assessed using a sequential batch test. Aliquots of concentrated Sb(III) solution and diluted with deionized water were added to a 500-ml Erlenmeyer flask to a total volume of 200 ml and 0.80 g Fe_3_O_4_/HCO was then added to the mixture and pH was adjusted using either 0.1 mol/L HCl or 0.1 mol/L NaOH solution. Adsorption was conducted at 150 rpm at temperature of 25 °C. After the reaction reached equilibrium, samples were filtered (0.45μm filter), and the concentration of Sb(III) determined in solution using hydride generation atomic fluorescence spectrometry (see below). All experiments were completed in triplicate and the adsorption tests were performed in the dark^31^. The effects of variation in pH (2–9), adsorbent dose (2.0–12.0 g/L), reaction time (2–24 h) as well as temperature (20, 25 and 30 °C) on Sb(III) removal were investigated.

For sorption isotherm experiments, 0.4 g of Fe_3_O_4_/HCO adsorbent was added to 100 mL Sb(III) solution (concentration range 10 to 200 mg/L). The initial pH of Sb(III) solution was 7.0 ± 0.1. After shaking at 150 rpm for 4 h, the residual Sb concentration in water was mensurated as above. All experiments were completed in triplicate at three temperatures (20 °C, 25 °C and 30 °C). Langmuir, Freundlich as well as Dubinin-Radushkevich (D-R) model (Eqs (S1–S5)) were used to fit to the Sb(III) adsorption data.

For the sorption kinetics experiments, 0.4 g portions of Fe_3_O_4_/HCO were added to 100 mL of 10, 50 100 mg/L Sb(III) solution. The initial pH of Sb(III) solution was 7.0 ± 0.1. The mixtures were shaken at 150 rpm at temperature of 25 °C. Subsequently 4 ml samples were taken at the following time intervals (10, 20, 30, 40, 50, 60, 90, 120, 150, 240, and 360 min) and the residual Sb concentration in solution determined. The method for the calculation of adsorption capacity is shown in Eq. (1).

1 $${\rm{q}}=\frac{({c}_{i}-{c}_{e})V}{1000M}$$

In which *q* (mg/g) is the adsorption capacity; *c*_*i*_ (mg/L) and *c*_*e*_ (mg/L) are the ion concentrations of the solution before and after the reaction, respectively; *V* (mL) is the volume of the solution; *M* (g) is the adsorbent mass used during the reaction process. In order to analyse the adsorption mechanism, four classic kinetic models, named as the Pseudo-first-order model, Pseudo-second-order model, Elovich model and Intra-particle diffusion model^30,32,33^ (Eqs (S6–S9)), were used to test fit to the experimental data.

### Reagents and analytical methods

A quantity of antimony potassium tartrate was weighed and dissolved in deionized water to prepare antimony standard bulk solution of 1.0 g/L Sb (III). Experimental Sb solutions were obtained by appropriate dilution. The reagents used in the experiment are analytical or superior grade reagents, and the experimental water was deionized water.

Hydride generation atomic fluorescence spectrometry was utilized to determine the concentrations of antimony (III), following the method of Leuz A *et al*.^34^. The detection limit of this method was 1 μg/L. All samples were measured within 24 h after the adsorption experiment, and deionized water was used for the blank. The Sb(III) recovery using this test was over 90.0% and analytical error on Sb determination was <1%.

## Results and Discussion

### Characterization of Fe_3_O_4_/HCO

**Figure 1:**
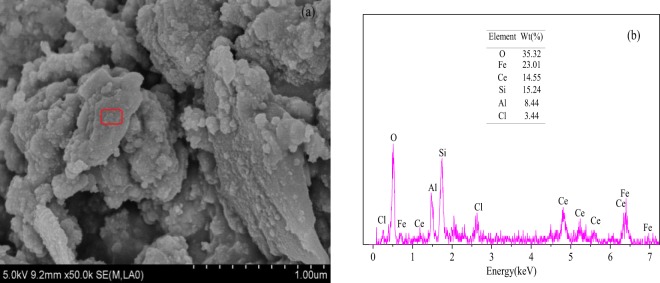
SEM image (**a**) and EDS (**b**) of Fe_3_O_4_/HCO sorbent.

The SEM-EDS characterization of Fe_3_O_4_/HCO shows that the surface was rough and irregular shapes from spherical (Fig. 1a). This suggested that amorphous substances were precipitated on the surface and the doping of the polishing sludge hampered the precipitation during the synthesis and changed the form of the desired products^17^. As shown in the Fig. 1b, the chemical composition of the Fe_3_O_4_/HCO adsorbent from EDS were O (35.32%), Fe (23.01%), Ce (14.55%), Si (15.24%), Al (8.44%) and Cl (3.44%), suggesting that the main components of the adsorbent are Fe and Ce oxides. In our study, the Ce/(Fe + Ce) molar ratio of Fe_3_O_4_/HCO was only about 0.20, which influences the surface area (S_BET_) and adsorption performance of Fe_3_O_4_/HCO^17,26^. This is slightly lower than the theoretical value (0.26) and the average reported values (about 0.23) by Qi *et al*.^17^ and Zhang *et al*.^26^. Further work is needed to evaluate the potential to synthese Fe_3_O_4_/HCO with higher Ce/(Fe + Ce) molar ratio.

The detailed XRD characterization of the sorbent is given in the supplementary data (Fig. S1). Ce and Fe and mixed oxide phases are identified. The peaks were observed at 2θ = 26.62°, 28.06°, 33.11°, 47.49°, and 56.40°, represent reflections from the (220), (311), (400), (511), and (440) planes, is different from other studies^17^. Qi^17^ *et al*. found that the doping Ce^III^ does not modify the original spinel structure of Fe_3_O_4_. The most probable reason for the deviation here is that the raw materials we used (particularly the sludge derived HCO) are different. The Fe_3_O_4_/HCO product contains discrete phases of Fe_3_O_4_, HCO and FeCe_2_O_4_, indicating a complex reaction between Fe_3_O_4_ and HCO occurred during the preparation. According to the structural characteristics of Fe_3_O_4_ and HCO, we speculated that FeCe_2_O_4_ could be synthesized from Fe_3_O_4_ and HCO by a double decomposition reaction (Fe_3_O_4_ + HCO + OH^−^ → FeCe_2_O_4_ + H_2_O) under the alkaline conditions during the preparation of the adsorbent. The synthesis of FeCe_2_O_4_ might play a major role in the adsorption of Sb(III) and its identification is an important innovation in this study.

The specific surface area (S_BET_) and pore volume of the Fe_3_O_4_/HCO was found to be 83.496 m^2^/g and 0.098 cm^3^/g, respectively. It was three times greater than the original Fe_3_O_4_ (S_BET_ 28.0 m^2^/g) and was much larger than hydrated ferric oxides supported by polymeric anion exchange^35^, indicating that the new sorbent has a significantly higher accessible surface area for adsorption of Sb(III).

### Effect of pH, react time, amount of adsorbent, temperature and initial concentration

**Figure 2:**
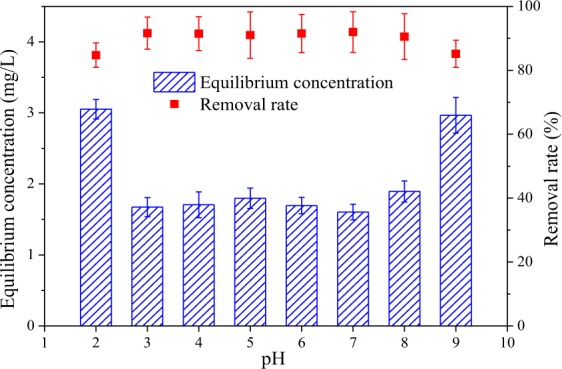
Effect of pH on the removal of Sb(III) ions by Fe_3_O_4_/HCO sorbent.

The pH and point of zero charge (pH_pzc_) are the most important parameters affecting Sb removal efficiency in adsorption technology^6,29^. As shown in Fig. 2, the removal of Sb(III) by Fe_3_O_4_/HCO varies with pH. The rate of Sb(III) removal initially increased in our experiment before decreasing as pH increased from 2 to 9, but only slightly changed (90.00–91.98%) over the pH 3 to 7 range. This was identified as optimal pH for Fe_3_O_4_/HCO to adsorb Sb(III). The measured zeta potentials of the Fe_3_O_4_/HCO suspensions was about 6.8, which was consistent with the literature results^17^. When the pH is 2, Sb(III) exists in the form of Sb(OH)_2_^+^ ^1^, competing with H^+^ and Sb(OH)_2_^+^ ^29^ which reduced the removal efficiency Sb(III). Across a pH range from 2 to 9, Sb(III) is predominantly in the form of [H_3_SbO_3_] or Sb(OH)_3_ ^1^ which can result in the precipitation of Fe-Sb(III)^36^ and CeSbO_3_ ^37^ with Fe_3_O_4_/HCO to enable a higher rate of Sb(III) removal. It was obvious that pH is close to the pH_pzc_ and makes the adsorbent surface uncharged and attracts the neutral Sb(OH)_3_. When pH > 9, oxidation of Sb(III) is enhanced^34^, and the increase in pH can inhibit the production of iron oxyhydroxide and the solubility of iron ions^18^, resulting in a decrease in the removal efficiency for Sb(III). These findings are consistent with those of Fan *et al*.^29^. Compared with other iron-based adsorbents^38,39^ (shown in Table 1), the adsorption of Sb(III) onto Fe_3_O_4_/HCO can occur over a wide pH, which introduces versatility and enhances potential application.

**Table 1:** Comparison of the published adsorption capacity for different sorbents with Sb(III) in water with results from this study.

Type of adsorbent	Initial concentration range (mg/L)	React time (h)	pH	Adsorbent dose (g/L)	Temperature (°C)	Adsorption capacity (mg/g)	Removal rate (%)	Reference
Hematite modified magnetic nanoparticles	0.11	2	4.1	0.1	25	36.7	>95.5	^41^
Fe(III)-loaded saponified orange waste	5.5	24	2.7	5	30	136.36	100	^42^
Zr(IV) and Fe(III) loaded orange waste	15	24	2.5	5	30	144.88	96	^42^
Iron-oxide coated sand	33.3	7	6	1.5	40	0.6	>95	^21^
FeCl_3_-modifed sepiolite	50	1.5	6.8	2	35	21.63	—	^52^
FeCl_3_-modifed activated carbon	1.5	—	5–9	1	25	—	—	^39^
Fe_2_O_3_ modified carbon nanotubes(CNTs)	1.5	2	7	0.5	25	6.3	99.97	^46^
Graphene oxide and it’s magnetite composites	0–150	2	3–9	1.2	25	8.7	>95	^38^
FeO(OH) modified clinoptilolite tuff	—	—	<2.7	—	20	7.17	—	^22^
Fe_2_O_3_-Fe_3_O_4_/C prepared with bamboo template	5–150	—	7	2	25	4.782	>90	^45^
Fe_2_O_3_-Fe_3_O_4_/C prepared with eucalyptus wood template	50	—	8	10	25	4.45	>90	^45^
Ce-doped_(0.5)_ Fe_3_O_4_	50	4.0	7.0	0.2	25	212.9	—	^17^
Fe_3_O_4_/HCO adsorbents	10–200	2	7.0	4	25	22.853	90–96	This study

**Figure 3:**
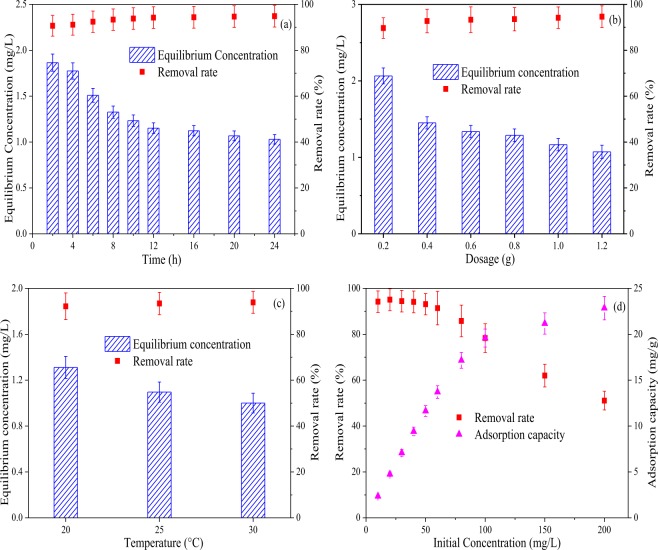
Effect of react time (**a**), amount of adsorbent (**b**), temperature (**c**) and initial concentration (**d**) on the removal of Sb(III) ions by Fe_3_O_4_/HCO sorbent.

The effect of reaction time on Sb(III) removal from water (Fig. 3a) suggests a two steps process: a fast stage from 0 to 2 h and a slow stage after 2 h. The reason for this is likely to be that initially surface adsorption takes place between 0–2 h. As the adsorption continues the binding sites on the adsorbent surface are saturated, therefore subsequent adsorption is by internal diffusion stage^40^ which reduced the rate of adsorption. The adsorption equilibrium time (2 h) of Fe_3_O_4_/HCO for removal Sb(III) is much less than that of many iron-loaded adsorbents, such as hematite coated magnetic nanoparticles^41^, iron (III) loaded orange peel residue^42^, and quartz sand loaded iron oxide^21^. The reaction equilibrium time of the subsequent experiments in this study was set to 2 h^43^.

Results for the effect of Fe_3_O_4_/HCO loading on Sb(III) removal (Fig. 3b) showed removal rates to increase from 89.68% to 92.75% with Fe_3_O_4_/HCO dosing from 2 g/L to 4 g/L. As the dose of Fe_3_O_4_/HCO rose to 12 g/L, the removal of Sb(III) slowly increased to 94.64%. This result indicates that Fe_3_O_4_/HCO loading has only a slight impact on the removal of Sb(III) when the addition of Fe_3_O_4_/HCO exceeds 4.0 g/L, a finding consistent with previous results of Sun,*et al*.^32,44^. As the adsorbent dosage increases, its surface can provide more functional groups and adsorption sites to enhance removal rate of Sb(III)^18^, while Sb(III) removal would not be improved when the adsorption equilibrium was reached. This indicates that an optimal dosage can be determined when other conditions remain stable^18^. Therefore, considering the cost of the adsorbent and the removal efficiency of Sb(III), the optimum loading of Fe_3_O_4_/HCO in our study was 4.0 g/L, this being less than that of iron (III) loaded orange peel residue (5 g/L)^42^, iron (III) and zirconium (IV) loaded orange peel residue (5 g/L)^42^ and composite material of biomorphic Fe_2_O_3_/Fe_3_O_4_/C with eucalyptus(10 g/L)^45^. This has positive practical application.

The effect of temperature shows a modest increase in sorption with increasing temperature. As shown in Fig. 3c, the removal of Sb(III) by Fe_3_O_4_/HCO adsorbent was 92.18%, 93.41% and 94.32% at 20 °C, 25 °C and 30 °C, respectively. The finding is consistent with studies of Sb(III) removal using ferric salts or ferric salt modified adsorbents^18,32^. Due to an endothermic reaction occurring when metal ions were adsorbed by the iron matrix, adsorption and removal efficiency, adsorption capacity and adsorption rate of most iron-based inorganic adsorbents will improve with an increase in temperature^35^. By taking operating costs into account, the optimal reaction temperature for this study was 25 °C.

As shown in Fig. 3d, an initial increase in concentration of Sb(III) from 10 mg/L to 60 mg/L resulted in a decrease in the removal of Sb(III) from 95.08% to 91.39%. When concentration increased to 200 mg/L, a significant decreased of Sb(III) removal to 51.10% was observed. This identifies good operating conditions at Sb(III) concentrations less than 60 mg/L. In addition, when the initial concentration of Sb(III) ranged from 150 mg/L to 200 mg/L, the adsorption sites on the Fe_3_O_4_/HCO sorbent were saturated when the initial concentration of Sb(III) was 200 mg/L^29^.

A comparison of the adsorption capacity of different iron-loaded composites for Sb(III) from water is shown in Table 1. The adsorption capacity of Fe_3_O_4_/HCO synthesized in this study is lower than the values of some adsorbents, such as Ce-doped_(0.5)_^17^, hematite coated magnetic nanoparticles^41^, iron (III) loaded orange peel residue^42^, and iron (III) and zirconium (IV) loaded orange peel residue^42^. However, it is significantly higher than a number of other adsorbents, for instance, ferric chloride modified sand^21^ and iron oxide loaded carbon nanotubes^46^. Also, the equilibrium time for Sb(III) adsorption Fe_3_O_4_/HCO for was only 2 h, considerably shorter than many other adsorbents.

### Adsorption isotherms

**Figure 4:**
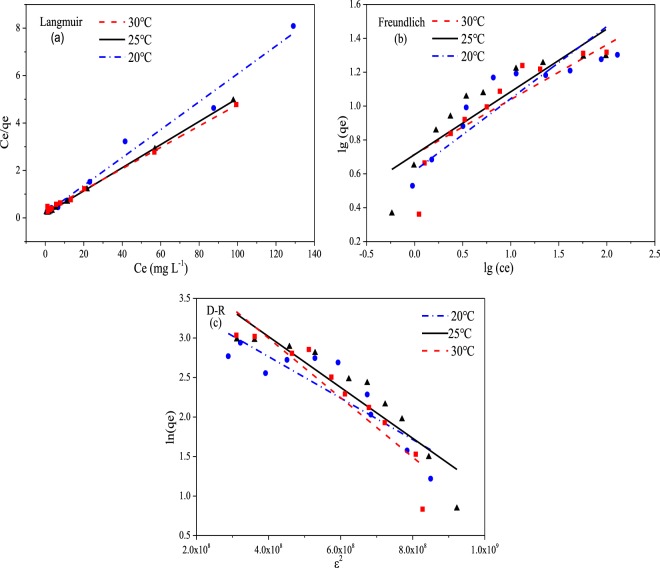
The Langmuir (**a**), Freundlich (**b**) and D-R (**c**) isotherms models for adsorption of Sb(III) onto Fe3O4/HCO sorbent at 20 °C, 25 °C and 30 °C.

Model definition and accuracy of fit for the isothermal adsorption model is related to the type of adsorbent, the valence state of the Sb ion, the initial concentration, pH, and a number of other factors^18,29,32^. The results for the fit to Langmuir, Freundlich and D-R model are shown in the Fig. 4. The parameters of the three adsorption isotherm models are listed in the Table 2.

**Table 2:** Isotherms parameters and correlation coefficients of different adsorption isotherm models for the adsorption of Sb(III) onto Fe3O4/HCO sorbent.

Models	Langmuir	Frundlich	D-R
Parameters	*q*_max_ = 23.171 mg/g	*K*_*f*_ = 5.108	*β* = −3.335E^−9^ mol^2^/KJ^2^
	*b* = 0.209 L/mg	1/n = 0.387	*q*_s_ = 80.743 mg/g
	*R*^2^ = 0.9996	*R*^2^ = 0.8214	*E* = 122.474 mol/KJ
			*R*^2^ = 0.8744

As shown in Fig. 4a, the linear relationship between *C*_e_/q_e_ and *C*_e_ at 20 °C, 25 °C and 30 °C indicates that the Langmuir model has strong fit at each temperature (R^2^ > 0.99). The maximum adsorption capacity and b value for Sb(III) at 25 °C were 23.171 mg/g and 0.209 L/mg, respectively.

As shown in Fig. 4b, a linear relationship between lg(*C*_*e*_) and lg(*q*_*e*_) for the Freundlich model at 20 °C, 25 °C and 30 °C was a slightly poorer fit (R^2^ > 0.82). The values of *K*_*f*_ as well as *1⁄n* are related to the adsorbent, adsorption mechanism and reaction temperature, which can be calculated by the relationship between lg(*C*_*e*_) and lg(q_e_). The isothermal adsorption form can be determined according to the value of *1⁄n*^31^. At 25 °C the *K*_*f*_ and *1⁄n* was 5.108 and 0.387, respectively. As the value of *1⁄n* was less than 0.5, which indicated that Sb(III) was easily adsorbed by Fe_3_O_4_/HCO^32^. This is also illustrated that Fe_3_O_4_/HCO is an excellent adsorbent for adsorbing antimony.

The linear relationship between *q*_*e*_ and *ε*^2^ of the D-R model at 20 °C, 25 °C and 30 °C are shown in Fig. 4c. The value of *q*_*s*_ and *β* values at 25 °C was 80.743 mg/g and 3.335E^−9^ mol^2^/KJ^2^, respectively. In addition the average adsorption energy *E* (kJ/mol), which could be determined from the D-R model, is the free energy change as one mole of ions transfers from the solution to the sorbent surface^47^. Using Eq. (S4), *E* ranged from 113.283 to 138.145 kJ/mol at 20–30 °C. According to the scale of the force and the *E* value between the adsorbed substance and the adsorbent, the adsorption process can be classified as physical adsorption (1 kJ/mol ≤ *E* ≤ 8 kJ/mol), ion exchange (9 kJ/mol ≤ *E* ≤ 16 kJ/mol) as well as chemical adsorption (*E* > 16 kJ/mol)^48^. Therefore, the adsorption of Sb(III) to Fe_3_O_4_/HCO was a chemisorption process^29^, which is also consistent with the conclusions by Deng *et al*. for adsorption of Sb(III) using Fe(III)-modified humus sludge^32^.

In summary, the Langmuir model has the best fit (R^2^ > 0.99) for the removal of Sb(III) by Fe_3_O_4_/HCO. This is consistent with the adsorption of Sb(III) by iron-based matrices^18,29,30,32^. We conclude that sorption reactions take place on the surface of iron-based adsorbents, as monolayer adsorption^29^. The Langmuir model obtained the maximum adsorption capacity of Fe_3_O_4_/HCO for removing Sb(III) (up to 23.171 mg/g at 25 °C), higher than many iron-based adsorptive substrates^21,46^. All of the *1/n* values was determined using the Freundlich model were less than 0.5, indicating that Sb(III) in an aqueous solution is readily adsorbed by Fe_3_O_4_/HCO^32^. Results for the D-R model further indicate that Sb(III) adsorption by Fe_3_O_4_/HCO is a chemisorption process^41^.

### Adsorption kinetics

**Figure 5:**
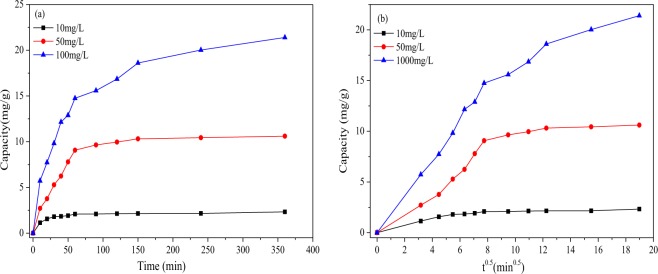
The fit of pseudo-first-order, pseudo-second-order, Elovich (**a**) and intra-particle diffusion (**b**) models of adsorption for Sb(III) onto Fe_3_O_4_/HCO.

The adsorption kinetics model can describe the potential rate of control and adsorption mechanism of material transfer and chemical reactions during the adsorption process. Adsorption kinetics are dominated by the physical as well as chemical properties of the adsorbent in the adsorption process of the adsorbent^11^. In this study, Pseudo-first-order, Pseudo-second-order, Elovich and Intra-particle diffusion models are used to analyze the kinetic characteristics of Fe_3_O_4_/HCO adsorption to remove Sb(III)^30,32,33^. As shown in Fig. 5a and Table 3, although a high correlation coefficient (R^2^ = 0.971) was recorded for the Pseudo-first-order model curve, the fitting of the curve tail was poor, indicating that the Pseudo-first-order model is not an appropriate simulation for Sb(III) adsorption onto Fe_3_O_4_/HCO. However, the Pseudo-second-order model showed the strongest fit with the experimental data (R^2^ = 0.993) in all adsorption kinetics. In addition, there is little difference between the theoretical value of *q*_*e*_(23.145 mg/g) and the experimental value (21.396 mg/g). Therefore, it is proposed that the kinetics of adsorption of Sb(III) by Fe_3_O_4_/HCO can be more accurately described by the Pseudo-second-order model^32^.

**Table 3:** A summary of parameters for the adsorption of Sb(III) onto Fe_3_O_4_/HCO in this study.

Models	Initial Sb(III) concentrations (mg/L)		
	10	50	100
Pseudo-first-order model	q_e_ = 2.134 mg/g	q_e_ = 10.614 mg/g	q_e_ = 19.688 mg/g
	K_1_ = 0.065 min^−1^	K_1_ = 0.025 min^−1^	K_1_ = 0.022 min^−1^
	R^2^ = 0.9774	R^2^ = 0.9887	R^2^ = 0.9705
Pseudo-second-order model	q_e_ = 2.323 mg/g	q_e_ = 12.361 mg/g	q_e_ = 23.145 mg/g
	k_2_ = 0.044 g/(mg.min)	k_2_ = 0.002 g/(mg.min)	k_2_ = 0.001 g/(mg.min)
	R^2^ = 0.9943	R^2^ = 0.9685	R^2^ = 0.9931
Elovich model	α = 3.343 mg/(mg.min)	α = 0.854 mg/(mg.min)	α = 1.487 mg/(mg.min)
	β = 3.438 g/mg	β = 0.399 g/mg	β = 0.216 g/mg
	R^2^ = 0.9652	R^2^ = 0.9314	R^2^ = 0.9916
Intra-particle diffusion model	α_1_ = 0.149 mg/g	α_1_ = −0.397 mg/g	α_1_ = −0.139 mg/g
	k_41_ = 0.278 mg/(g. min^0.5^)	k_41_ = 1.064 mg/(g. min^0.5^)	k_41_ = 1.855 mg/(g. min^0.5^)
	R^2^_1_ = 0.9410	R^2^_1_ = 0.9657	R^2^_1_ = 0.9938
	α_2_ = 1.909 mg/g	α_2_ = 8.432 mg/g	α_2_ = 10.176 mg/g
	k_42_ = 0.020 mg/(g. min^0.5^)	k_42_ = 0.126 mg/(g. min^0.5^)	k_42_ = 0.616 mg/(g. min^0.5^)
	R^2^_2_ = 0.8088	R^2^_2_ = 0.7739	R^2^_2_ = 0.9507

In addition, results for the fit to the curve between *q*_*t*_ and ln*t* (Fig. 5a) and the calculated parameters (Table 3) highlight that Elovich model also has a good fit with the experimental data (R^2^ = 0.992)^31^.

The intra-particle diffusion model describes the diffusion relationship between the adsorbate and the pores of the adsorbent^29,49^. As shown in Fig. 5b, the fit of the plots of *q*_*t*_ and *t*^0*.5*^ can be divided into two steps, including a fast initial and slow later adsorption stages. The difference between the slopes of the first and second phases, indicate that a gradual phase exists in which the surface adsorption process is controlled by thickness of the boundary layer. The two intercepts (*α*1, *α*2) represent the thickness of the theoretical boundary layer of the two stages. As shown in Table 3, the significant difference between *α*1(−0.139) and *α*2(10.176) demonstrates that the pore diffusion rate is not a unique control process. Therefore, the adsorption rate of Sb(III) by Fe_3_O_4_/HCO is determined by the boundary layer effect and the external mass transfer effect^29,31^.

### Thermodynamic studies

Gibbs free energy ΔG° (kJ/mol), standard enthalpy change ΔH° (kJ/mol) as well as standard entropy change ΔS° (J/(mol.k)) are the main parameter of adsorption thermodynamics. In the study, Eqs (2) and (3) were used to calculate the ΔG°, ΔH^0^ and ΔS^0^ for Sb(III) adsorption onto Fe_3_O_4_/HCO at 20 °C, 25 °C as well as 30 °C (Table 4). Adsorption properties were also investigated, as well as its spontaneity.

2 $$\Delta {G}^{0}=-\,{{\rm{RTlnK}}}_{0}$$

3 $$\mathrm{ln}\,{K}_{{\rm{0}}}=\frac{\varDelta {S}_{{\rm{0}}}}{R}-\frac{\varDelta {H}_{{\rm{0}}}}{RT}$$

**Table 4:** The thermodynamic calculation results for adsorption of Sb(III) on Fe_3_O_4_/HCO.

Thermodynamic parameters	Temperature(K)		
	293.15	298.15	303.15
K_0_	4.062	5.108	5.183
ΔG^0^ (Kj/mol)	−3.183	−3.704	−3.736
ΔH^0^ (kJ/mol)	33.427	—	—
ΔS^0^ (J/(mol.k))	11.75	—	—

In which *R* is the molar constant, 8.314 J/(mol.k); *T* is the absolute temperature, K; *K*_0_ is the equilibrium constant of adsorption thermodynamics. *K*_*0*_ can acquire according to the method stated by Zheng^47^ and the Freundlich equation was used to fit the parameters to calculate *K*_0_, namely, *K*_0_ = *Kf*.

As shown in Table 4, the value of *K*_0_ increased (from 4.062 to 5.183) as temperature increased (from 293.15 K to 303.15 K), suggesting that the adsorption process of Sb(III) on Fe_3_O_4_/HCO was an endothermic reaction^50^. The values of ΔG^0^ at 293.15 K, 298.15 K and 303.15 K were −3.183 kJ/mol, −3.704 kJ/mol and −3.736 kJ/mol, respectively. All ΔG^0^ less than 0 indicates that adsorption of Sb(III) on Fe_3_O_4_/HCO was a spontaneous process^50^. Furthermore, the decrease of ΔG^0^ with temperature increasing implied that the degree of spontaneous adsorption could be enhanced with increasing temperature. The values of ΔH^0^ and ΔS^0^ were 33.427 kJ/mol and 11.75 J/(mol.k), respectively. ΔH^0^ > 0 further indicates that the adsorption process is endothermic. ΔS^0^ > 0 demonstrates which adsorption occurred on the surface of the Fe_3_O_4_/HCO adsorbent as a process of random improvement on the solid-liquid surface, and the arrangement of the adsorbed Sb(III) on the surface of Fe_3_O_4_/HCO was chaotic, probably owing to the release of water molecules from hydrated Sb(III)^33^.

### Desorption

In this study, desorption of the adsorbed Sb(III) ions from sorbent was also studied in a series of batch experiments. The efficiencies of the different eluents are shown in Table 5. Compared with HCl and NaOH, the repetitive adsorption rate of Fe_3_O_4_/HCO to Sb(III) ions after EDTA and water repeatedly desorption was very low. In a 2 cycle adsorption-desorption process, after desorption by EDTA and pure water, the removal rate of Fe_3_O_4_/HCO to Sb(III) ions decreased to less than 80%(65.27% and 78.21%). After 3 and 4 cycles of adsorption-desorption process with HCl and NaOH desorption, the removal of Sb(III) ions by Fe_3_O_4_/HCO is still close to 80%(79.91% and 79.22%). Compared to HCl, NaOH is cheaper and safer. Thus NaOH solution was used as a desorption agent. Sb(III) ions desorption from Fe_3_O_4_/HCO created the removal process economical both adsorbent and Sb(III) ions were regenerated and recycled effectively.

**Table 5:** Effects of stripping agents on Sb(III) desorption.

Stripping agents	Time (h)	Temperature (°C)	Cycle	Removal rate (%)
H_2_O	2	25	2	78.21
0.1 mol/L NaOH	2	25	4	79.22
0.1 mol/L HCl	2	25	3	79.91
0.1 mol/L EDTA	2	25	2	65.27

### Adsorption mechanism

**Figure 6:**
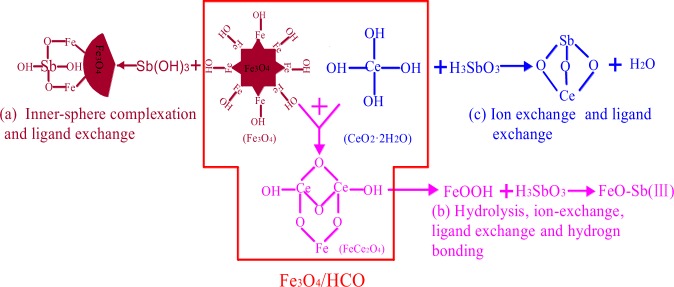
The adsorption mechanism proposed for Sb(III) onto Fe_3_O_4_/HCO.

From the characterization of before and after the adsorption of Sb on the Fe_3_O_4_/HCO, the mechanisms proposed for the Sb(III) adsorption on Fe_3_O_4_/HCO are illustrated in Fig. 6. The possible reactions in the adsorption process are speculated as Eqs (4)~(8). And the preferred adsorption mechanisms between Sb(III) and Fe_3_O_4_/HCO was concluded as following:

4 $${\rm{X}}\equiv \mathrm{Fe}-\mathrm{OH}+{\rm{Sb}}{({\rm{OH}})}_{3}\to {\rm{X}}\equiv \mathrm{Fe}-\mathrm{Sb}{({\rm{OH}})}_{2}+{{\rm{H}}}_{2}{\rm{O}}$$

5 $${\rm{X}}\equiv \mathrm{Fe}-({{\rm{CeO}}}_{2}\cdot {{\rm{nH}}}_{2}{\rm{O}})+{{\rm{H}}}_{3}{{\rm{SbO}}}_{3}\to {\rm{X}}\equiv \mathrm{Fe}-({{\rm{CeSbO}}}_{3})+({\rm{n}}+1){{\rm{H}}}_{2}{\rm{O}}$$

6 $${{\rm{FeCe}}}_{2}{{\rm{O}}}_{4}+{{\rm{H}}}_{2}{\rm{O}}\to {\rm{FeOOH}}+{{\rm{CeO}}}_{2}+{{\rm{OH}}}^{-}$$

7 $${\rm{FeOOH}}+{{\rm{H}}}_{3}{{\rm{SbO}}}_{3}\to {\mathrm{FeO}-H}_{2}{{\rm{SbO}}}_{3}+{{\rm{H}}}_{2}{\rm{O}}$$

8 $${{\rm{Sb}}}^{3+}+2{{\rm{Ce}}}^{4+}={{\rm{Sb}}}^{5+}+2{{\rm{Ce}}}^{3+}$$

1. Adsorption of Sb by Fe_3_O_4_. Firstly, the Fe_3_O_4_/HCO may hydrolyze iron octahedron, inner sphere complexes and other spherical complexes^36^. Subsequently, Sb(III) can preferentially interact with the A-type hydroxyl group of iron octahedron in Fe_3_O_4_ to form a monodentate mononuclear, monodentate dinuclear or bidentate dinuclear ligand via the aforementioned ligand-exchange reaction^20,51^ (Fig. 6a). In addition, Sb(III) may be adsorbed by the inner sphere complex of Fe_3_O_4_ and other spherical complexes^36^ (Eq. (4)).
2. Adsorption of Sb by HCO. When the pH of aqueous solution is 6.7, HCO is a hydrated metal oxide with zero surface charge. The XRD diffraction pattern (Fig. S2) confirms that the compound CeSbO_3_ exists in the residual precipitate after adsorption. Therefore, the second reaction mechanism of Fe_3_O_4_/HCO adsorbing Sb(III) is speculated as shown in Fig. 6b. CeSbO_3_ was synthesized by the reaction of HCO with H_3_SbO_3_(HCO + H_3_SbO_3_ → CeSbO_3↓_ + H_2_O), and the main mechanism of HCO adsorbing anions in water is the exchange reaction of anionic ligands^37^ (Eq. (5)).
3. FeCe_2_O_4_ was used to hydrolyze HCO and FeOOH, and then they reacted with Sb (III). In the preparation of Fe_3_O_4_/HCO, two Ce^3+^ ion replaced Fe^3+^ at octahedral sites in a lattice structure (Fe_3_O_4_ + HCO + OH^−^ = FeCe_2_O_4_ + H_2_O). FeCe_2_O_4_ was hydrolyzed in aqueous solution, and electron and ion was transferred occurred between phase interface and aqueous solution, forming a double-electron layer structure. This results in the *in situ* formation of an amorphous hydrated iron oxide which has a larger specific surface area^19^. Ligand exchange as well as adsorption of Sb (III) occurred on the iron oxide film (Eqs (6)~(7)).
4. From other studies, it can be concluded that Ce(IV) itself being a good oxidizing agent (Ce^4+^/Ce^3+^ = 1.72) in acidic medium^27^. As shown in the Fig. S4, Ce(IV) can oxidizes the surface sorbed Sb(III) to Sb (V) (Eq. (8)), It is basically consistent with the Sb 3d XPS Spectra. And Ce(IV) itself getting reduced to Ce^3+^ according to the underlying redox reaction.

## Conclusion

Results from our study indicate that Fe_3_O_4_/HCO is a novel efficient and environment-friendly sorbent for removal the Sb(III) from wastewater. The pH, adsorbent loading, temperature and initial concentration of the Sb(III) solution all effect the adsorbent ability of Fe_3_O_4_/HCO. When pH was between 3–7, Sb(III) was readily removed from the solution. The maximum adsorption capacity for Fe_3_O_4_/HCO adsorbing Sb(III) was 22.8534 mg/g. Compared with the Freundlich as well as D-R models, the Langmuir model had a highest fitting accuracy for Sb(III) adsorption to Fe_3_O_4_/HCO. After fitting the adsorption data with different kinetic models, the Pseudo second-order model is most suitable. The ΔG^0^ and ΔH^0^ values indicate that the adsorption of Sb(III) by Fe_3_O_4_/HCO is a spontaneous endothermic process. The ΔS^0^ > 0 indicates an increase in entropy during adsorption. The mechanism of the adsorption of Sb(III) on Fe_3_O_4_/HCO can be defined by synergistic adsorption onto discrete Fe_3_O_4_, FeCe_2_O_4_ and HCO phases.

## Supplementary information

Supplementary Information
