# Supplementary material for: Preparation of a novel Fe3O4/HCO composite adsorbent and the mechanism for the removal of antimony (III) from aqueous solution
Source: Sci Rep. 2019 Sep 10;9:13021. doi: 10.1038/s41598-019-49679-9 (PMC6737065; doi:10.1038/s41598-019-49679-9)
Supplement: Supplementary file 1 — Supplementary Information [file 41598_2019_49679_MOESM1_ESM.docx]

Preparation of a novel Fe_3_O_4_/HCO composite adsorbent and the mechanism for the removal of antimony (III) from aqueous solution

Jun Zhang^1^, Ren-jian Deng^1,2^*, Bo-zhi Ren^1^, Baolin Hou^1^, Andrew Hursthouse^1,2,3^

*^1^School of Civil Engineering, Hunan University of Science and Technology, Xiangtan 411201, China*

^2^*Hunan Jing Yi Environmental Protection High Tech Development Co. Ltd., Xiangtan, China*

^3^*School of Computing, Engineering & Physical Sciences, University of the West of Scotland, Paisley PA1 2BE, UK*

**Corresponding author at:School of Civil Engineering,Hunan University of Science and Technology, Xiangtan 411201, China. Tel.: +086 8773255885, E-mail address:deng800912@163.com*

Drawings

Figure S1. XRD Phase identification for Fe_3_O_4_/HCO adsorbent

Figure S2. XRD Phase identifying for Fe_3_O_4_/HCO adsorbent with adsorbed Sb(Ⅲ)

Figure S3. XPS broad scan for Fe_3_O_4_/HCO adsorbent after adsorption Sb(Ⅲ)

Figure S4. XPS fine scan of Fe_3_O_4_/HCO adsorbent after adsorption for Sb(Ⅲ)

Equation

**Equation S1**

The Langmuir isotherm model assumes that the adsorption processes of the adjacent adsorption sites do not affect each other, and all adsorption sites on the surface of adsorbent have equal solute affinity. This model has a monolayer adsorption^29^, and its isothermal adsorption equation is expressed as equation (S1).

(S1)

In which q_e_ (mg/g) and q_max_ (mg/g) are adsorption capacity and maximum adsorption capacity, respectively; b (L/mg) is the equilibrium constant related to adsorption energy, the properties of the adsorbent and the reaction temperature, and a higher b value represents a stronger adsorption performance of the adsorbent; and (mg/L) is the Sb(III) concentration in solution at the equilibrium of adsorption.

**Equation S2**

Freundlich isotherm model assumes that adsorption sites on the surface of the adsorbent are non-uniform, this being suitable for monolayer and multilayer adsorption. The adsorption mechanism of monolayer or multilayer adsorption is mainly determined by the solution concentration and temperature-dependent adsorption rate^32^. The linear expression of the Freundlich model is:

$\lg\left( q_{e} \right)=lg\left( K_{f} \right)+\frac{1}{n}\lg\left( C_{e} \right)$ lg(q_e_)=lg(K*_f_*)+1/nlg(C_e_) (S2)

where, *C_e_* (mg/L) is equilibrium concentration of Sb(III) in the solution; *K_f_* and *1⁄n* are the constants of the Freundlich model. The values of *K_f_* as well as *1⁄n* are related to the adsorbent, adsorption mechanism and reaction temperature, which can be calculated by the relationship between lg(*C_e_*) and lg(q_e_). The isothermal adsorption form can be determined according to the value of *1⁄n*^31^. *1⁄n* value from 0.1 to 0.5 indicates that the solute is easily adsorbed by the adsorbent, while *1⁄n* > 2.0 indicates that the solute is difficult to be adsorbed by the adsorbent. In addition, *1⁄n* > 1 or *1⁄n* < 1 represents normal adsorption or synergistic adsorption, respectively^32^.

**Equation S3-S5**

The D-R isotherm assumes that the adsorbent has a constant adsorption potential or a non-uniform surface^29^. Moreover, the linear relationship of the D-R model can be used to predict whether the adsorption of Sb(III) by Fe_3_O_4_/HCO is a physical adsorption or chemical adsorption process. The linear relationship of the D-R model was calculated as equation (S3).

(S3)

where, β (mol^2^/kJ^/2^) is the D-R model constant; q_s_ (mg/g) is isothermal saturation capacitance in theory; and is the polanyi adsorption potential, which can be calculated as formula (S4).

(S4)

In which R is the universal gas constant, 8.314 J/(mol.K); T is the absolute temperature (298.15 K in this study); *C_e_*(mol/L) is the Sb(III)concentration in the solution at the equilibrium of adsorption. Both of *q_s_* and *β* can be identifid according the relationship between and .

Additionally, the average adsorption energy *E* (kJ/mol), which could be determined according the D-R model, is the free energy change as one mole of ions transfers from the solution to the sorbent surface^48^. It can be calculated as equation (S5).

(S5)

According to the scale of the force and the *E* value between the adsorbed substance and the adsorbent, the adsorption process can be classified into physical adsorption (1 kJ/mol≤*E*≤8 kJ/mol), ion exchange (9 kJ/mol≤*E*≤16 kJ/mol) as well as chemical adsorption (*E*>16kJ/mol) ^49^.

**Equation S6**

The Pseudo-first-order model can be described as equation (S6) .

(S6)

where, ( min^-1^) is the adsorption rate constant; (mg/g) and (mg/g)are the adsorption capacities at *t* min and adsorption equilibrium, respectively.

**Equation S7**

The Pseudo-second-order model assumes that the adsorption rate is controlled by chemisorption, which is related to the binding force existing in the electron sharing or electron transfer between the adsorbate and the adsorptive matrix^32^. This model can be described as equation (S7).

(S7)

where, (g/mg.min) is the adsorption rate constant of the Pseudo-second-order model; (mg/g) is the adsorption capacity; (mg/g) is the adsorption capacity at *t* min.

**Equation S8**

Elovich model was originally used to describe the kinetics of gas adsorption in solids and was subsequently used to depict the adsorption kinetics of solids in water^32^. This model can be describes as:

(S8)

In which (mg/g) is the adsorption capacity at *t* min; (mg/(mg.min) is the adsorption constant of Sb(III); and(g/mg) is a constant relate to the surface area of the adsorbent and the chemical activation energy.

**Equation S9**

The adsorption rate of the porous material is largely determined by the intra-particle diffusion coefficients of the adsorbate. The intra-particle diffusion model describes the diffusion relationship between the adsorbate and the pores of the adsorbent^29, 50^, which can be expressed as:

(S9)

where, (mg/g.min^0.5^) is the adsorption rate constant at a certain stage of adsorption, which is determined by the thickness of the boundary layer; is the constant of the Intra-particle diffusion model at a certain adsorption stage, reflecting the significance of the mass transfer between the boundary layer and the external^29^. It is generally believed that the greater the intercept, the stronger the boundary layer effect^29^. The adsorption capacity is linearly dependent to *t^0.5^*. The fitting straight line of the intra-particle diffusion model passes through the origin, indicating that the adsorption process is mainly controlled by the internal diffusion of particles. If the fitted curve exhibits a multilinear relationship, this indicates that the adsorption process includes two or more adsorption steps^29^.
